# Supplementary material for: Splice-Junction-Based Mapping of Alternative Isoforms in the Human Proteome
Source: Cell Rep. Author manuscript; Available in PMC 2020 Jan 15. (PMC6961840; doi:10.1016/j.celrep.2019.11.026)
Supplement: 3 [file NIHMS1546469-supplement-3.zip › DF2/PXD006675/PulmonaryValve-42-P22105-VGPVSAVGVTAPGK.pdf]

A

Predicted sequence disorder and sequence features of P22105

Peptide: VGPVSAVGVTAPGK Junction: sp|P22105|TENX_HUMAN|ENSG00000168477|SE2|40039|chr6|32062483|32065117|−2|r5|T1 TrNovel: TRUE

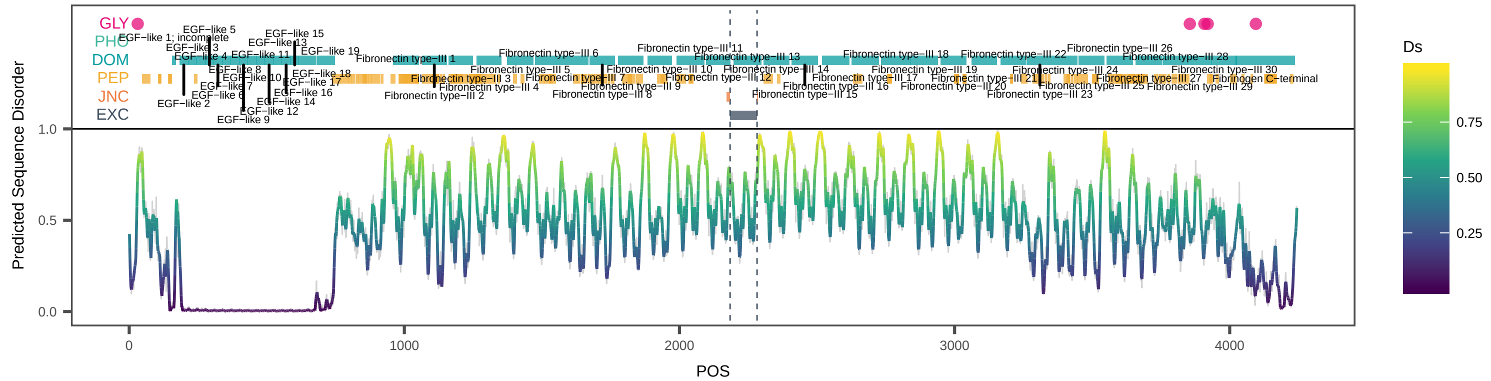

B

Distribution of sequence disorder in excised vs. mapped and non−excised regions of protein

M−W P−value vs. mapped: 0.000618 vs. non−excised: 0.0168

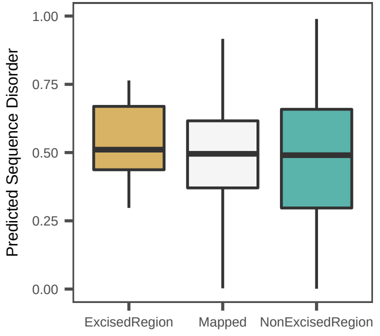

C
